# PW02-009 - PAPA syndrome: results from the Euroefever registry

**DOI:** 10.1186/1546-0096-11-S1-A149

**Published:** 2013-11-08

**Authors:** R Caorsi, A Insalaco, D Marotto, J Frenkel, A Martini, F De Benedetti, M Gattorno

**Affiliations:** 1 2nd Division Of Pediatrics, Istituto Gaslini, Genoa, Genova, Italy; 2 Department of Pediatrics, Ospedale Pediatrico Bambino Gesù, Rome, Italy; 3 Department of Rheumathology, Ospedale di Tempio Pausania, Tempio Pausania (OT), Italy; 4 Department of Pediatrics, University Medical Center, Utrecht, The Netherlands

## Introduction

PAPA syndrome is a very rare autoinflammatory condition. Few data are nowadays available about the clinical characteristic, the response to treatment and the outcome of this disease.

## Objectives

To analyse the data of the PAPA patients enrolled to the Eurofever registry.

## Methods

The data analysed in the study were extracted from the Eurofever registry, which is hosted in the PRINTO website (http://www.printo.it). The patients were included in the study in the presence of clinical manifestations consistent with PAPA syndrome and mutations in the PSTPIP1 gene. Demographic data, clinical manifestations and response to treatment were analysed.

## Results

In February 2013 baseline and clinical information were available of 2567 patients from 88 centers in the Eurofever registry. Of these 16 patients PAPA patients (M:F = 8:8), from 3 different centers, fulfilled the inclusion criteria and were therefore analysed: 10 were of the same family, in 3 patients the disease was caused by a *de novo* mutation while in 3 cases the mutation was found in one parent (not yet included in the registry). The mean age at enrolment was 26,22 years (4 paediatric and 12 adult patients). The mean age at disease onset was 5,7 years (range birth – 18 years). The mean age at diagnosis was 24,5 years (range 1,8 – 57,5), with a mean delay of 18,8 years (range 2 months – 50 years). The mutations found in the PSTPIP1 gene were V344I (1pt), E250K (1 pt), E257G (1 pt), A230T (2 pts), and E250Q (11 pts).

The disease course was recurrent in 8 patients, while the other 8 presented a chronic disease course with periodic recrudesces. 15 patients presented an articular involvement during their disease course, while 11 patients presented clinical manifestations affecting the skin (folliculitis in 8, pyoderma gangrenosum in 3, skin abscess 8 patients) and 2 patients complained with suppurative hidradenitis. 7 out of the 16 patients presented clinical manifestations not typical of PAPA syndrome (psoriasis, osteolytic bone lesions, chronic renal failure, muscular abscesses, anaemia and hepatosplenomegaly). Response to treatment with NAIDS was as partial or absent in 8 and 2 patients respectively, while the steroids caused a complete or partial control of disease manifestations in 5 and 6 patients respectively. Two patients were treated with methotrexate with partial response. Etanercept was used in one patient with complete response, adalimumab in 3 patients (2 partial and 1 complete responders) and anakinra in 4 patients (2 partial and 2 complete responders).

## Conclusion

The study analyses the largest series of PAPA syndrome patients described so far. The wide clinical heterogeneity and the usual presentation with a single manifestation might be responsible for under-recognition of the syndrome.

## Disclosure of interest

None declared.

